# MicroRNA-mediated regulation of cell wall dynamics and intercellular communication under stress

**DOI:** 10.3389/fpls.2026.1871374

**Published:** 2026-06-24

**Authors:** Ana Belén Mendoza-Soto, Katia Aviña-Padilla

**Affiliations:** 1 Facultad de Estudios Superiores Iztacala, Universidad Nacional Autónoma de México, Tlalnepantla, Mexico; 2 Department of Cell Biology, Centro de Investigación y de Estudios Avanzados del Instituto Politécnico Nacional (CINVESTAV-IPN), Mexico City, Mexico

**Keywords:** cell wall remodeling, microRNAs, plant cell wall, post-transcriptional regulation, stress response

## Abstract

MicroRNAs (miRNAs) are short endogenous non-coding RNAs that regulate gene expression at the post-transcriptional level through mRNA cleavage or translational repression. In plants, miRNAs play pivotal roles in a wide range of biological processes, including development, growth, and responses to biotic and abiotic stresses. Central to these processes is the plant cell wall, a dynamic structure that not only provides mechanical support but also functions as the first line of defense against environmental challenges and pathogen attack. The plant cell wall must maintain a fine balance between rigidity and plasticity, enabling rapid remodeling in response to developmental cues and stress signals. This remodeling process requires tight and coordinated regulation of genes involved in cell wall biosynthesis, modification, and degradation. Emerging evidence indicates that miRNAs are key regulators of these processes, modulating the expression of transcription factors, enzymes, and signaling components associated with cell wall dynamics. In addition to their intracellular roles, miRNAs can also function as mobile signals, linking cell wall remodeling with intercellular communication through plasmodesmata and coordinating responses at the tissue level. Furthermore, recent studies suggest that pathogen-derived small RNAs may contribute an additional regulatory layer by targeting host genes associated with cell wall structure and connectivity. In this review, we summarize recent advances in the identification and functional characterization of miRNAs involved in the regulation of plant cell wall responses. We highlight their regulatory implications, target genes, and roles in integrating developmental, stress-related, and intercellular communication pathways. Understanding the RNA-mediated control of cell wall dynamics provides new insights into plant adaptability and resilience, with potential applications in crop improvement and stress tolerance engineering.

## 1 Introduction

Plant microRNAs (miRNAs) are central regulators of gene expression that operate predominantly at the post-transcriptional level, modulating a wide spectrum of developmental and physiological processes. These small non-coding RNAs, typically 21 nucleotides in length—although 22- and 24-nucleotide variants contribute to functional diversification—mediate gene silencing through sequence-specific recognition of target mRNAs, resulting in transcript cleavage or translational repression (Voinnet, 2009; Axtell et al., 2011; Vidigal and Ventura, 2015; Song et al., 2019). Through these mechanisms, miRNAs function as fine-tuning regulators that shape gene expression landscapes in response to developmental cues and environmental signals. In plants, miRNAs are embedded within complex regulatory networks that integrate hormonal signaling, metabolic pathways, and responses to biotic and abiotic stress. Beyond their well-established roles in controlling transcription factors and signaling cascades, emerging evidence indicates that miRNAs also influence the structural and functional dynamics of the plant cell wall. This expanded regulatory scope positions miRNAs as key mediators at the interface between gene regulation, cellular architecture, and environmental adaptation. (Zhan and Meyers, 2023).

miRNAs are encoded by MIR genes and transcribed by RNA polymerase II into primary transcripts (pri-miRNAs) characterized by stem–loop structures. These transcripts undergo nuclear processing mediated by a microprocessor complex composed of DICER-LIKE 1 (DCL1), HYPONASTIC LEAVES 1 (HYL1), and SERRATE (SE), which sequentially generate precursor miRNAs (pre-miRNAs) and miRNA/miRNA* duplexes (Dong et al., 2008; Xie, 2015). The duplex is subsequently methylated by HUA ENHANCER 1 (HEN1), ensuring stability, and exported to the cytoplasm via HASTY (Li et al., 2005). There, the guide strand is incorporated into an ARGONAUTE (AGO) protein to form the RNA-induced silencing complex (RISC), which directs gene silencing through near-perfect complementarity with target transcripts. Importantly, miRNA biogenesis and activity are dynamically regulated by developmental and environmental signals, enabling context-dependent modulation of gene expression.

This regulatory plasticity is particularly relevant in processes that require rapid cellular reprogramming, such as cell wall remodeling. The plant cell wall is not a static structure but a highly dynamic and responsive matrix that must continuously adjust its composition and mechanical properties to balance rigidity and plasticity during growth and stress responses. In addition to providing mechanical support, the cell wall serves as a critical interface for environmental perception, defense activation, and intercellular communication.

Recent advances have shown that miRNAs contribute to these processes by modulating the expression of transcription factors, biosynthetic enzymes, and signaling components involved in cell wall organization and remodeling. Through these regulatory networks, miRNAs influence key processes such as lignification, polysaccharide modification, cellulose deposition, and plasmodesmatal function, thereby linking structural dynamics with signaling pathways. Furthermore, the ability of miRNAs to act as mobile signals suggests that they may coordinate cell wall responses across tissues, integrating local and systemic adaptation to environmental challenges.

In addition to endogenous miRNAs, emerging evidence indicates that pathogen-derived small RNAs may introduce an additional regulatory layer by targeting host genes associated with cell wall structure and intercellular connectivity. Together, these findings support a model in which RNA-mediated regulation plays a central role in coordinating cell wall dynamics with developmental programs, stress responses, and intercellular communication.

In this review, we summarize current advances in the identification and functional characterization of miRNAs involved in the regulation of plant cell wall responses. We highlight their molecular mechanisms, target pathways, and roles in integrating developmental, stress-related, and communication processes. Understanding how RNA-mediated regulatory networks shape cell wall dynamics provides new insights into plant adaptability and resilience. Given the central role of the cell wall as both a structural and signaling interface, the following section examines its composition, organization, and function as a dynamic platform for intercellular communication.

## 2 The plant cell wall as a dynamic interface for intercellular communication

The plant cell wall is a highly dynamic and multifunctional structure that not only provides mechanical support but also plays a central role in signaling and intercellular communication. It is primarily composed of cellulose microfibrils embedded within a matrix of hemicelluloses and pectins, with lignin contributing to rigidity in specialized tissues, thereby defining cell shape and mechanical properties (Cosgrove, 2005; Bidhendi and Geitmann, 2016; Vaahtera et al., 2019). Two major types of cell walls can be distinguished: the primary cell wall, which is flexible and extensible to accommodate cell growth, and the secondary cell wall, which is deposited after expansion and is typically thicker and lignified, providing enhanced structural reinforcement (Cosgrove, 2005). Beyond its structural role, the cell wall establishes the apoplast, an extracellular continuum that facilitates the diffusion of ions and signaling molecules. In parallel, the symplast constitutes a cytoplasmic network interconnected by plasmodesmata (PD), enabling direct cell-to-cell communication. The coordination between apoplastic and symplastic pathways is essential for regulating transport, signaling, and developmental patterning across tissues (Sattelmacher, 2001). Importantly, the cell wall is not a passive barrier but an active signaling interface, where cell wall integrity is continuously monitored by receptor-like kinases that integrate developmental and environmental cues (Wolf et al., 2012; Vaahtera et al., 2019).

The close association between the cell wall and plasmodesmata establishes a direct link between wall composition and symplastic connectivity. Plasmodesmata are embedded within the cell wall matrix, and their permeability is dynamically regulated by the deposition and degradation of callose at their neck regions, which modulates the movement of macromolecules between cells (De Storme and Geelen, 2014; Knox and Benitez-Alfonso, 2014). Consequently, changes in cell wall composition and mechanics directly influence intercellular trafficking and communication (Amsbury et al., 2017; Wu et al., 2018.

Far from being a static barrier, the cell wall undergoes continuous remodeling mediated by cell wall-modifying factors, including expansins, non-enzymatic proteins that disrupt non-covalent interactions between cellulose and hemicellulose to facilitate cell expansion (Cosgrove, 2005, 2022); pectin methylesterases, which regulate the degree of pectin methylesterification and thereby influence wall stiffness and porosity (Wolf et al., 2012); and peroxidases, which participate in oxidative cross-linking reactions that can either stiffen or reinforce the wall depending on the cellular context, modulating wall extensibility, porosity, and mechanical strength (Passardi et al., 2004). Cell wall remodeling also involves hydrolytic and de-esterifying enzymes, including glycoside hydrolases, β-glucanases, xyloglucan endotransglucosylase/hydrolases (XTHs), polygalacturonases, pectate lyases, acetylesterases, and pectin acetylesterases. These enzymes remodel cellulose–hemicellulose and pectin networks, alter wall porosity and stiffness, and participate in stress-induced wall loosening or reinforcement (Bayer and Benitez-Alfonso, 2024). These modifications are essential not only for growth and differentiation but also for adaptive responses to environmental stimuli, where rapid changes in wall composition influence both apoplastic diffusion and plasmodesmatal permeability. In this context, the plant cell wall emerges as a central regulatory hub that integrates structural dynamics with intercellular communication and environmental responsiveness. Given this central role, the regulation of cell wall dynamics requires finely tuned gene expression programs. Plant miRNAs have emerged as key post-transcriptional regulators capable of modulating pathways associated with cell wall biosynthesis, remodeling, and differentiation. Although miRNAs do not function as structural cell wall components, they regulate transcripts encoding wall-associated enzymes, transcription factors, and signaling components that control polymer deposition, modification, or degradation (Rogers and Chen, 2013; Achkar et al., 2016).

Several miRNA-mediated modules illustrate this regulatory connection. Copper-responsive miRNAs, particularly miR397, miR408, and miR857, target laccase transcripts involved in monolignol oxidation and polymerization, thereby linking nutrient/redox homeostasis with secondary wall reinforcement (Bologna and Voinnet, 2014; Wang et al., 2014a). In addition, mobile miRNAs such as miR165/166 contribute to spatial patterning during vascular development by establishing transcriptional gradients that indirectly shape cell wall properties and tissue organization (Carlsbecker et al., 2010). Together, these findings highlight that miRNA-mediated regulatory networks are tightly coupled to the control of cell wall composition and mechanics, positioning small RNAs as critical modulators of plant structural plasticity and intercellular communication.

Importantly, the integration of cell wall remodeling with plasmodesmatal regulation suggests that intercellular connectivity is not solely determined by structural properties but also by dynamic molecular signaling networks. In this context, the ability of miRNAs to move between cells introduces an additional regulatory layer, linking gene expression control with the spatial coordination of cell wall dynamics across tissues. This highlights the need to understand how small RNA mobility is modulated within the cell wall environment.

## 3 MicroRNA-mediated regulation of cell wall dynamics

Cell wall remodeling is governed by coordinated changes in polymer biosynthesis, modification, degradation, and cross-linking. Although miRNAs do not function as structural cell wall components, they regulate transcripts encoding wall-associated enzymes, transcription factors, and signaling proteins that shape wall composition and mechanics. Direct regulatory examples include miRNAs targeting laccases involved in lignin polymerization, whereas indirect modules act through HD-ZIP III, MYB, SPL, ARF, and TCP transcription factors that coordinate vascular differentiation, phenylpropanoid metabolism, hormone responses, and growth–defense trade-offs (Ralph et al., 2004; Vanholme et al., 2010; Schuetz et al., 2014). This distinction between direct enzymatic regulation and indirect developmental control is essential for interpreting miRNA effects on cell wall dynamics.

**Figure 1:**
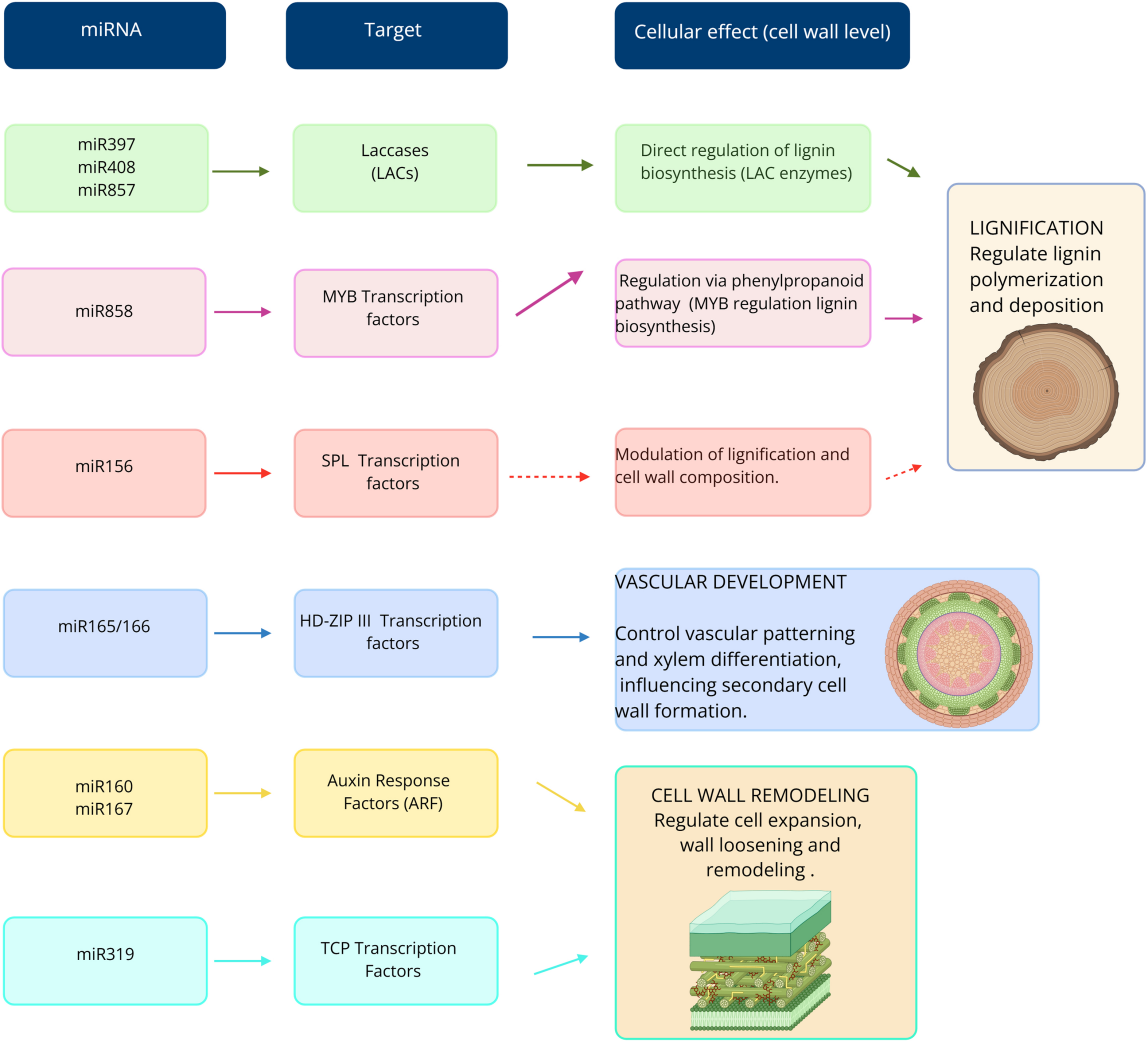
MicroRNA-mediated regulation of plant cell wall dynamics. Environmental and developmental cues regulate plant microRNAs (miRNAs), which in turn control target genes involved in cell wall-related processes. Copper-responsive miRNAs (miR397, miR408, and miR857) directly target laccases to regulate lignin polymerization. Other miRNAs act indirectly through transcription factors, including HD-ZIP III (miR165/166), MYB (miR858), SPL (miR156), AUXIN RESPONSE FACTORS (miR160 and miR167), and TCPs (miR319). These interactions converge on lignification, vascular development, and cell wall remodeling. Solid and dashed arrows indicate direct and indirect or context-dependent effects, respectively.

As shown in **Figure 1**, Copper-responsive miRNAs, particularly miR397, miR408, and miR857, regulate lignification primarily by targeting laccase (LAC) transcripts involved in monolignol oxidation and polymerization. Laccases are multicopper oxidases that catalyze the oxidative coupling of monolignols into lignin, a key phenylpropanoid-derived polymer that confers rigidity and mechanical strength to secondary cell walls (Boerjan et al., 2003; Abdel-Ghany et al., 2008). This module links nutrient/redox homeostasis with secondary wall reinforcement and becomes especially relevant under stress conditions, where altered lignin deposition can modify wall rigidity, pathogen resistance, and barrier properties. Through post-transcriptional repression of LAC transcripts, these miRNAs modulate lignin deposition: miR397-mediated repression of laccases has been associated with reduced lignification and altered secondary cell wall properties, whereas its downregulation under stress conditions promotes lignin accumulation and wall strengthening (Wang et al., 2014; Zheng et al., 2019). In rice and *Beckmannia syzigachne*, miR397 has also been linked to oxidative stress responses and herbicide tolerance through the regulation of laccase-associated antioxidant pathways, highlighting its role at the interface between redox homeostasis and cell wall remodeling (Pan et al., 2017).

Several miRNAs influence cell wall properties indirectly by targeting transcription factors that specify vascular identity, hormone responses, or developmental phase transitions. These include miR165/166–HD-ZIP III, miR858–MYB, miR156–SPL, miR160/167–ARF, and miR319–TCP modules. miR858 modulates MYB transcription factors involved in the phenylpropanoid pathway, thereby influencing the balance between flavonoid production and lignification (Sharma et al., 2016). Auxin-related miRNAs such as miR160 and miR167 regulate AUXIN RESPONSE FACTOR (ARF) transcription factors, which control cell expansion and growth-related processes and have been associated with the expression of genes involved in cell wall loosening (Liu et al., 2007; Zheng et al., 2019).

miR319 regulates TCP transcription factors that coordinate cell proliferation and expansion, thereby indirectly influencing cell wall remodeling and organ growth (Palatnik et al., 2003; Koyama et al., 2010; Wang et al., 2014b). The miR156–SPL module has been associated with increased cellulose accumulation and reduced lignin content, as observed in alfalfa, suggesting a role in modulating the balance between primary and secondary wall components (Gao et al., 2016).

Together, these findings indicate that miRNA-mediated regulation of the plant cell wall operates through a multilayered network integrating direct enzymatic control with developmental and hormonal regulation, ultimately shaping cell wall composition, structure, and function. These regulatory processes are tightly coupled to intercellular communication pathways, reinforcing the concept that cell wall dynamics and RNA-mediated signaling operate as an integrated system. Beyond these global regulatory roles in cell wall remodeling, miRNAs also exert precise spatial control over cell wall properties, contributing to the establishment of developmental barriers and tissue patterning. **Table 1** summarizes these miRNAs, their target genes, and their cell wall-related functions.

**Table 1:** MicroRNAs associated with plant cell wall dynamics: target genes and cell wall-related functions.

miRNA	Target genes	Biological process	Cell wall-related function	Type of regulation	Reference
miR397	Laccases (LAC genes)	Lignin biosynthesis	Controls lignification and secondary cell wall formation	Direct	Wang et al., 2014; Zheng et al., 2019
miR408	Laccases, copper proteins	Redox balance, lignification	Modulates lignin deposition	Direct	Pan et al., 2018; Guo et al., 2023
miR857	Laccases (e.g., LAC7)	Lignin biosynthesis	Regulates lignin polymerization and secondary wall formation	Direct	Abdel-Ghany et al., 2008; Zhao et al., 2015.
miR858	MYB transcription factors	Phenylpropanoid pathway	Regulates genes involved in lignin and flavonoid biosynthesis	Indirect	Sharma et al., 2016
miR165/166	HD-ZIP III TFs	Vascular development	Controls xylem differentiation and cell wall patterning	Indirect	Carlsbecker et al., 2010.
miR156	SPL transcription factors	Developmental timing	Affects cell wall composition during phase transitions.	Indirect	Gao et al., 2016.
miR319	TCP transcription factors	Leaf and stem development	Influences cell expansion, cell elongation and cell wall synthesis	Indirect	Schommer et al., 2008; Sun et al., 2017; Cao et al., 2020
miR160	ARF transcription factors	Auxin signaling	Impacts cell wall formation and wall plasticity	Indirect	Liu et al., 2007.
miR167	ARF6/8	Auxin signaling, cell expansion	Associated with the regulation of cell wall loosening genes and cell expansion	Indirect	Wu et al., 2006

TFs, transcription factors; ARF, AUXIN RESPONSE FACTOR; HD-ZIP III, Homeodomain-leucine zipper class III; LAC, laccase; SPL, SQUAMOSA Promoter-Binding Protein-Like; TCP, TEOSINTE BRANCHED1/CYCLOIDEA/PCF.

In addition to developmental regulation, stress-responsive miRNAs contribute to the dynamic control of cell wall properties under environmental challenges. miR408 has been associated with regulatory networks linked to carbohydrate metabolism, metal ion binding, and cell wall-associated responses during cold stress, suggesting a potential role in adaptive wall remodeling (Ma et al., 2015, Lu et al., 2005). Likewise, miR398, a key regulator of reactive oxygen species (ROS) homeostasis, may indirectly influence cell wall modification through redox-dependent processes affecting lignin polymerization and polysaccharide cross-linking (Sunkar et al., 2006; Wolf et al., 2012).

## 4 MicroRNAs in the regulation of developmental barriers and tissue patterning

Beyond their role in general cell wall remodeling, miRNAs are essential regulators of spatial patterning and the formation of developmental barriers that define tissue organization. These processes rely on the precise control of gene expression gradients that ultimately determine cell wall composition and mechanical properties in specific cell types.

One of the best-characterized examples is the miR165/166–HD-ZIP III regulatory module, which establishes positional information during root and vascular development. Mobile miR165/166 gradients restrict HD-ZIP III expression to specific domains, thereby controlling xylem differentiation and the spatial deposition of secondary cell walls (Carlsbecker et al., 2010). This gradient-based regulation is critical for defining vascular patterning and ensuring proper mechanical strength and conductive function of plant tissues.

miRNAs also contribute to the formation of specialized apoplastic barriers, such as the endodermis and the Casparian strip, which regulate selective transport and protect internal tissues from environmental fluctuations. Through the regulation of transcription factors, including members of the MYB and NAC families, miRNAs indirectly influence the expression of genes involved in lignin and suberin deposition, key components of these barriers. These modifications determine wall impermeability, mechanical strength, and the regulation of nutrient and water fluxes across tissues.

Furthermore, the spatial control exerted by miRNAs integrates developmental programs with environmental inputs, allowing plants to dynamically adjust tissue architecture under changing conditions. By modulating both transcriptional regulators and cell wall-associated enzymes, miRNAs coordinate the establishment of structural barriers and tissue-specific wall properties, contributing to the overall organization and functionality of plant organs. Importantly, these spatially regulated processes are closely linked to intercellular communication, as the mobility of specific miRNAs enables the formation of gradients that coordinate cell wall differentiation across tissues. This highlights the integration of RNA-mediated signaling with cell wall dynamics in shaping plant development at the tissue and organ levels.

When plant miRNAs are produced in the nucleus, they are exported to the cytosol, where they associate with AGO proteins to form RISCs that mediate post-transcriptional gene regulation. Beyond their intracellular function, a distinctive feature of plant miRNAs is their ability to act as mobile signaling molecules, enabling communication between neighboring cells and distant tissues.

In plants, intercellular connectivity is mediated by plasmodesmata (PD), plasma membrane-lined nanochannels that traverse the cell wall and establish cytoplasmic continuity between adjacent cells. Primary PD are formed during cytokinesis, whereas secondary PD arise across existing cell walls and often exhibit greater structural complexity. These channels create a symplastic continuum that allows the movement of water, ions, proteins, and RNA molecules, including small RNAs (Kitagawa and Jackson, 2017).

Importantly, PD permeability is tightly regulated by the deposition of callose, a β-1,3-glucan polymer that accumulates at the PD neck region. Callose synthesis by callose synthases restricts PD aperture, whereas its degradation by β-1,3-glucanases restores intercellular connectivity. This dynamic balance provides a biophysical gating mechanism that controls the intercellular trafficking of signaling molecules, including miRNAs (Kitagawa and Jackson, 2017; Tee and Faulkner, 2024). Consequently, the mechanical properties of the cell wall—particularly callose turnover—directly determine PD conductivity and, therefore, the mobility of miRNAs.

Several studies have demonstrated that miRNAs can move between cells in a non-cell-autonomous manner. In the *Arabidopsis thaliana* shoot apical meristem (SAM), miR394 is expressed in the L1 epidermal layer but moves into the underlying L2 and L3 layers, where it represses *LEAF CURLING RESPONSIVENESS* (LCR) and contributes to stem cell maintenance. More broadly, small RNAs, including miRNAs, siRNAs, and tasiRNAs, can act as mobile morphogens, forming gradients that define developmental domains and coordinate tissue patterning (Dong et al., 2022).

The movement of small RNAs occurs at multiple spatial scales. At the local level, miRNAs move cell-to-cell through plasmodesmata, whereas at the systemic level they can be transported via the vascular system, particularly through the phloem, enabling long-distance signaling between organs. However, not all miRNAs are equally mobile; accumulating evidence indicates that mobility is selective and regulated, depending on sequence features, structural properties, and interactions with RNA-binding proteins (De Meo et al., 2025; Lin et al., 2026). In many cases, mobile small RNAs are transported as duplexes or in association with specific protein complexes that stabilize and guide their movement.

The cell-wall environment plays a central role in modulating miRNA mobility. The structural composition of the wall, particularly the abundance and modification of pectins, hemicelluloses, and lignin, can influence PD architecture and permeability. Changes in pectin cross-linking or wall stiffness may alter PD density and aperture, thereby affecting symplastic connectivity. In addition, stress-induced remodeling of the cell wall frequently leads to callose accumulation, which restricts PD-mediated transport and may limit the spread of miRNAs as part of defense or compartmentalization responses.

Beyond the symplastic pathway, alternative routes of miRNA transport have been proposed. Extracellular vesicles (EVs) have emerged as potential carriers of small RNAs through the apoplast, capable of traversing the cell wall matrix and participating in both intra-plant communication and cross-kingdom RNA interference during plant–pathogen interactions (Wekesa et al., 2026). Although direct evidence for endogenous miRNA transport via EVs in developmental contexts remains limited, their selective loading and stability suggest an additional regulatory layer in RNA mobility.

Collectively, these findings indicate that miRNA movement in plants is governed by a complex interplay between plasmodesmatal gating, cell wall composition, and RNA transport mechanisms. The integration of the mechanical properties of the cell wall with molecular trafficking pathways represents a key regulatory axis in plant biology. In this framework, cell wall remodeling not only influences miRNA mobility but is itself shaped by RNA-mediated regulatory networks, reinforcing a bidirectional relationship between structural dynamics and intercellular signaling. Future advances combining live-cell imaging, spatial transcriptomics, and nanoscale analysis of cell-wall architecture will be essential to dissect how these processes coordinate the spatial dynamics of gene regulation mediated by miRNAs.

## 5 MicroRNAs, cell-wall remodeling and stress responses

**Figure 2:**
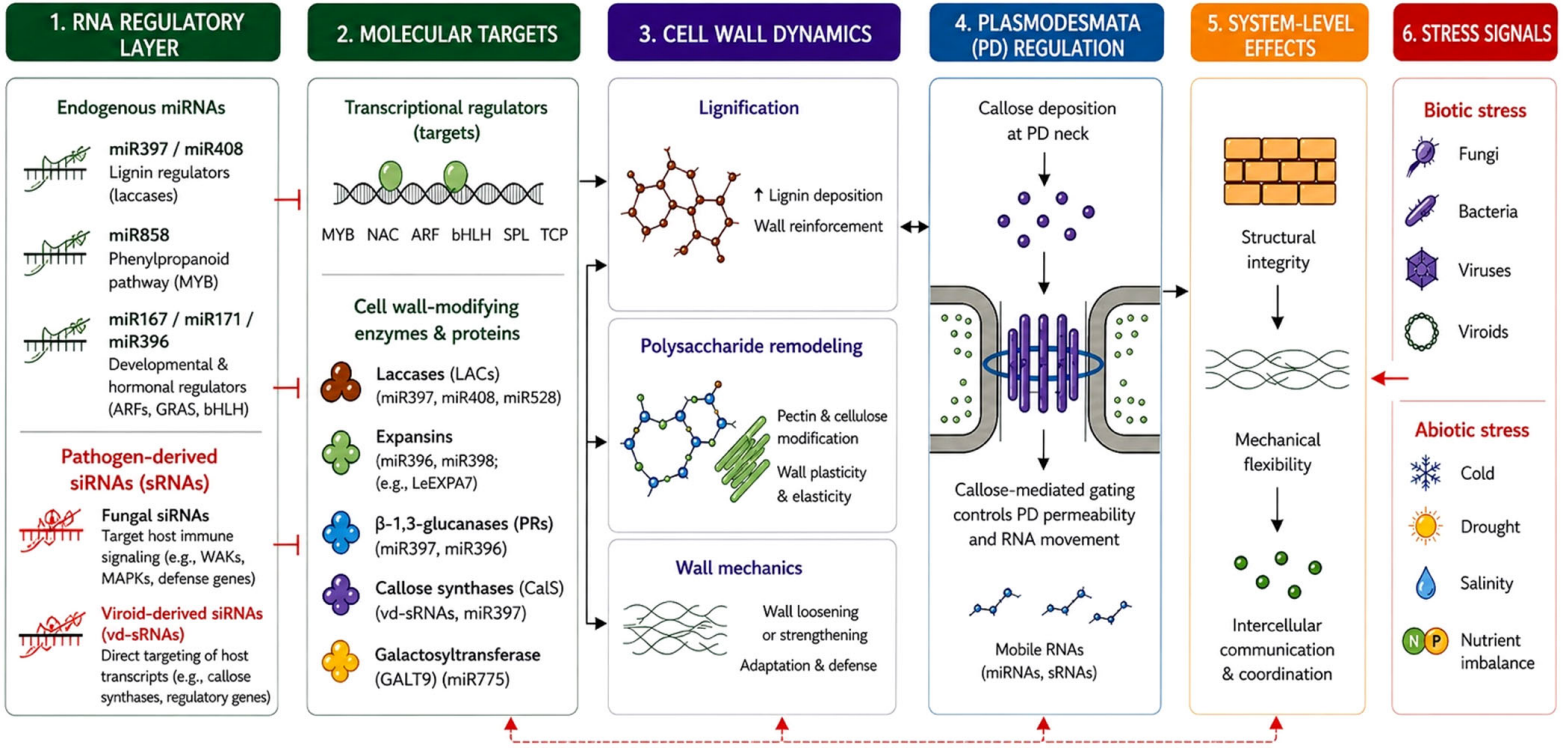
MicroRNA-mediated regulation of cell wall dynamics and plasmodesmatal connectivity under biotic and abiotic stress. Endogenous microRNAs (miRNAs) regulate transcription factors (e.g., SPL, TCP, ARF, GRAS, and bHLH/GRF) and cell wall–modifying enzymes, thereby controlling lignin, pectin, and cellulose biosynthesis and modulating cell wall architecture in response to stress. Through these regulatory cascades, miRNAs integrate developmental and stress-related signals to fine-tune cell wall remodeling, balancing structural integrity with cellular flexibility. In parallel, viroid-derived small RNAs (vd-sRNAs) provide an additional layer of regulation by directly targeting host transcripts, including callose synthases, thereby modulating callose deposition at plasmodesmata (PD) and influencing intercellular connectivity. Together, these RNA-mediated pathways contribute to the coordination of defense, growth, and adaptation under both biotic (fungi, bacteria, viruses, and viroids) and abiotic (cold, drought, salinity, and nutrient imbalance) stress conditions.

As illustrated in **Figure 2**, multilayered RNA-mediated regulatory networks integrate endogenous miRNAs, pathogen-derived small RNAs, and transcription factors to control cell wall remodeling and plasmodesmatal connectivity under stress conditions. Through these interconnected layers, plants dynamically adjust cell wall composition and mechanical properties in response to environmental challenges, balancing structural integrity with cellular flexibility and intercellular communication. These regulatory mechanisms operate across both biotic and abiotic stress contexts, where small RNAs fine-tune gene expression programs associated with defense, development, and adaptation. In the following sections, we examine how miRNA-mediated regulation differentially contributes to cell wall remodeling under biotic and abiotic stress conditions.

### 5.1 Biotic stress

During biotic stress, plant cell wall remodeling constitutes one of the earliest and most effective defense responses, integrating structural reinforcement with multilayered regulatory mechanisms at both transcriptional and post-transcriptional levels. Upon pathogen perception, plants rapidly induce callose deposition, lignification, phenolic cross-linking, and extensive reprogramming of cell wall-modifying enzymes, thereby enhancing wall rigidity and reducing permeability to restrict pathogen entry and spread (Underwood, 2012; Malinovsky et al., 2014). Callose is a dynamic cell wall glucan that, despite its low abundance, plays a central role in regulating plasmodesmata permeability and intercellular signaling during plant development and stress responses. Its accumulation modulates key processes such as phloem function, cell division, and defense against pathogens and environmental stressors, including toxic metals. However, the molecular mechanisms governing callose synthesis and degradation remain incompletely understood, representing a critical gap for improving plant resilience and interactions with beneficial and pathogenic organisms (German et al., 2023). Increasing evidence indicates that small RNAs, particularly miRNAs, play a central role in orchestrating these responses by fine-tuning gene expression networks associated with cell wall biosynthesis and remodeling.

Under pathogen challenge, modulation of the miR397–LAC module contributes to defense-associated lignification. This conserved regulatory axis directly links post-transcriptional regulation with lignin biosynthesis: laccases catalyze the oxidative polymerization of monolignols into lignin, a key determinant of cell wall strength. Across multiple plant–pathogen systems, repression of miR397 leads to increased LAC expression and enhanced lignification, thereby reinforcing the cell wall as a physical barrier against invasion (Lu et al., 2013; Wang et al., 2014; Wei et al., 2021, Yang et al., 2023). This mechanism should be interpreted as a context-dependent regulatory response rather than a universal outcome, as it has been observed in diverse contexts, including fungal infections such as *Verticillium dahliae* in cotton and *Macrophomina phaseolina* in chickpea, as well as in bacterial and viral interactions.

Beyond this core module, additional miRNA-mediated regulatory networks contribute to cell wall remodeling during infection. The miR858–MYB axis modulates the phenylpropanoid pathway, influencing the balance between flavonoid biosynthesis and lignification, while miR408 and miR528 target copper-binding proteins and laccases, integrating redox homeostasis with structural reinforcement. Similarly, miR160 and miR167 regulate ARFs, linking hormonal signaling with cell wall plasticity and growth–defense trade-offs (Winnicki et al., 2021). Collectively, these networks demonstrate that miRNAs integrate metabolic, hormonal, and structural pathways to coordinate adaptive responses.

Emerging evidence further expands this framework by incorporating plant–microbiome interactions. Small RNAs have been shown to influence rhizosphere dynamics and microbial associations, indirectly affecting cell wall-associated defense mechanisms. In this context, Mendoza-Soto et al. (2022) demonstrated that miR397 is induced in mycorrhizal tomato plants challenged with *Sclerotinia sclerotiorum*, suggesting its involvement in a primed defense state that enhances cell wall reinforcement. This finding highlights the integration of miRNA regulation with beneficial microbial interactions, extending beyond classical pathogen responses.

Another important layer of regulation involves cross-kingdom RNA communication, where small RNAs are exchanged between plants and interacting organisms. Plant-derived miRNAs can target pathogen virulence genes, while pathogen-derived small RNAs suppress host defense pathways, revealing a bidirectional regulatory system that influences both gene expression and structural defense responses (Ding et al., 2024). This mechanism further supports the role of small RNAs as mediators of inter-organismal communication.

Within this broader context, viroids provide a unique model of RNA-driven regulation. Unlike viruses, viroids do not encode proteins but generate abundant viroid-derived small RNAs (vd-sRNAs) that hijack the host RNA silencing machinery (Navarro et al., 2012; Adkar-Purushothama et al., 2015; Navarro et al., 2009; Aviña-Padilla et al., 2015). These vd-sRNAs can directly target host transcripts, including genes involved in cell wall biosynthesis and plasmodesmatal regulation. In *Potato spindle tuber viroid* (PSTVd)-infected plants, callose synthase transcripts have been experimentally validated as vd-sRNA targets, establishing a direct mechanistic link between RNA silencing, cell wall remodeling, and intercellular connectivity.

Because callose deposition at plasmodesmata regulates symplastic transport, vd-sRNA-mediated modulation of callose synthases may influence both defense signaling and pathogen movement. Increased callose accumulation restricts plasmodesmatal permeability, limiting pathogen spread but also affecting the movement of signaling molecules, including miRNAs (Zavaliev et al., 2011; De Storme and Geelen, 2014; Faulkner, 2018). Conversely, reduced callose levels may facilitate systemic signaling and pathogen dissemination. In this context, endogenous miRNAs and vd-sRNAs may interact within the same regulatory network, potentially competing or cooperating to shape gene expression and tissue-level responses.

In addition to vd-sRNA-mediated effects, viroid infection alters the accumulation of endogenous miRNAs that regulate developmental and hormonal pathways. In tomato infected with pospiviroids, miRNAs such as miR167, miR171, miR396, and miR408 are differentially expressed, targeting transcription factors including ARFs, GRAS, and bHLH proteins (Aviña-Padilla et al., 2018). These regulators orchestrate gene expression programs associated with cell expansion, differentiation, and structural remodeling.

Importantly, recent work on the regulatory role of bHLH transcription factors during PSTVd infection further supports the integration of transcriptional and post-transcriptional networks in controlling cell wall dynamics (Aviña-Padilla et al., 2022, 2025). These studies revealed tissue-specific reprogramming of bHLH-associated regulatory networks, with leaf responses enriched in defense-related pathways and root responses linked to growth modulation and adaptive remodeling. Such spatial coordination highlights the complexity of cell wall regulation during infection. The rhizosphere is a dynamic interface where plants interact with diverse biotic and abiotic factors, with miRNAs emerging as key regulators of plant adaptation and signaling. Through miRNA–mRNA crosstalk, plants modulate growth, development, and stress responses shaped by belowground environmental conditions (Fahad et al., 2025).

Taken together, current evidence supports a model in which small RNAs regulate cell wall remodeling during biotic stress through interconnected mechanisms that integrate enzymatic control, transcriptional regulation, and intercellular communication. Endogenous miRNAs modulate lignin biosynthesis and hormonal pathways, while pathogen-derived small RNAs directly target host genes involved in wall structure and plasmodesmatal regulation. This multilayered regulatory framework positions the plant cell wall as both a physical defense barrier and a dynamic signaling interface shaped by RNA-mediated networks during plant–pathogen interactions.

### 5.2 Abiotic stress

The regulatory role of miRNAs in cell wall dynamics extends beyond biotic interactions and is highly relevant under abiotic stress conditions, where maintaining cellular integrity and flexibility is essential for survival. Under cold or metal stress, miR397–LAC regulation appears to fine-tune lignin deposition and ROS-associated wall remodeling, with outcomes depending on species, tissue, and developmental stage. In *Brassica napus*, integrative transcriptomic and small RNA analyses have identified Bna-miR397a as a key regulator of BnaLAC2 under long-term low-temperature stress. Post-transcriptional repression of BnaLAC2 by Bna-miR397a reduces lignin remodeling and contributes to the maintenance of ROS homeostasis, a critical factor during cold stress. Functional studies further demonstrated that overexpression of Bna-miR397a enhances cold tolerance in both rapeseed and *Arabidopsis thaliana*, whereas its repression compromises freezing tolerance. These findings indicate that fine-tuning lignin biosynthesis through miR397-mediated regulation constitutes a conserved strategy for cold acclimation, balancing structural reinforcement with oxidative stress management (Hussain et al., 2025; Li et al., 2021). Cadmium exposure triggers robust, tissue-specific reprogramming of miRNA–mRNA regulatory networks in *Brassica napus*, involving differentially expressed miRNAs and hundreds of differentially expressed genes in roots and shoots, with anti-regulatory interaction pairs (e.g., miR398 targets) coordinating antioxidant defense, transcriptional activation, and key metabolic pathways such as glutathione and phenylpropanoid metabolism (Fu et al., 2019). Notably, Yang et al. (2024) showed that the miR397a–LAC17 module regulates lignin biosynthesis in Medicago ruthenica, while Pan et al. (2024) reported miR397-mediated cadmium responses through lignin polymerization in mangrove roots.

Beyond lignin-associated pathways, several miRNAs regulate additional components of the cell wall matrix. A notable example is miR775, a Brassicaceae-specific miRNA that targets the galactosyltransferase gene *GALT9*, involved in pectin biosynthesis. Overexpression of miR775 or loss of GALT9 function results in increased cell size and reduced cell wall stiffness, as demonstrated by biochemical assays and atomic force microscopy. Mechanistically, the transcription factor HY5 represses *MIR775A* expression, thereby promoting pectin accumulation and increased wall rigidity (Zhang et al., 2021). Although initially characterized in the context of organ growth, these changes in wall elasticity are highly relevant under abiotic stress, where increased compliance may enhance tolerance to desiccation, osmotic stress, or mechanical strain.

Genome-wide and transgenic studies further reveal additional miRNA modules that influence cell wall composition and stress adaptation. The miR156–SPL module has been associated with increased cellulose accumulation and reduced lignin content, thereby modulating the balance between primary and secondary wall components (Gao et al., 2016). Similarly, miR319–TCP modules regulate transcription factors involved in secondary growth; overexpression of miR319 in bioenergy crops delays lignification and improves biomass digestibility (Schwab et al., 2005). Another key regulator, miR408, targets copper-binding proteins including laccases and plastocyanin-like proteins; its overexpression in poplar reduces lignin accumulation and increases cell wall degradability, linking nutrient homeostasis with structural remodeling (Lu et al., 2013).

Collectively, these studies demonstrate that miRNAs act as central integrators of environmental cues, hormonal signaling, and metabolic pathways, enabling plants to dynamically adjust cell wall composition and mechanics under abiotic stress. Through coordinated regulation of lignin biosynthesis, pectin remodeling, and cellulose deposition, miRNA-mediated networks maintain a balance between structural integrity and adaptive flexibility. **I**mportantly, these regulatory processes are functionally linked to intercellular communication pathways, as changes in wall composition and mechanics can influence plasmodesmatal permeability and the mobility of signaling molecules, including small RNAs.

**Figure 3:**
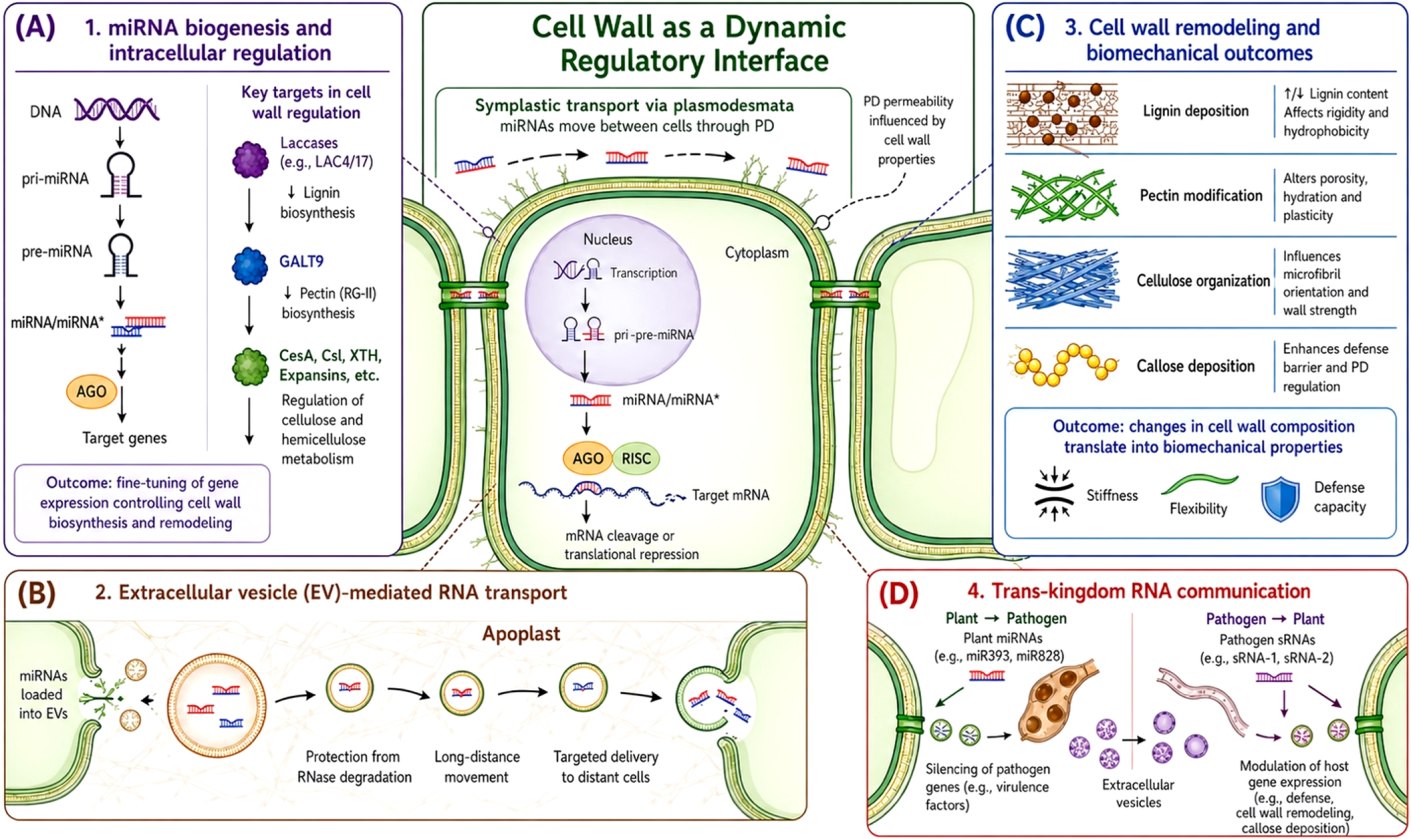
miRNA-mediated regulation of plant cell wall dynamics across multiple biological scales. **(A)** miRNA biogenesis and intracellular regulation, where miRNAs are processed and incorporated into the AGO/RISC complex to regulate target genes involved in cell wall biosynthesis, including lignin, pectin, and cellulose pathways. **(B)** Extracellular vesicle (EV)-mediated RNA transport, illustrating the loading of miRNAs into EVs and their movement through the apoplast, enabling protected and long-distance signaling. **(C)** Cell wall remodeling and biomechanical outcomes, showing how miRNA-mediated regulation affects lignin deposition, pectin modification, cellulose organization, and callose deposition, ultimately influencing stiffness, flexibility, and defense capacity. **(D)** Trans-kingdom RNA communication, where plant-derived miRNAs target pathogen genes and pathogen-derived small RNAs modulate host gene expression, including pathways associated with cell wall remodeling. Together, these processes position the plant cell wall as a dynamic regulatory interface integrating structural, molecular, and signaling networks.

Taken together, current evidence indicates that miRNAs regulate multiple components of the plant cell wall, including lignin, pectin, cellulose, and callose, through both direct targeting of biosynthetic enzymes and indirect modulation via transcription factors. This multilayered regulation integrates developmental and stress-responsive pathways, reinforcing the concept of the cell wall as a dynamic regulatory interface (**Figure 3**).

## 6 Perspectives and future directions

The integration of miRNA biology with plant cell wall dynamics is rapidly evolving into a conceptual framework that transcends classical gene regulation, positioning small RNAs as central players at the interface between structure, signaling, and adaptation. While earlier studies largely relied on correlative evidence linking miRNA expression with changes in lignin content or wall-associated gene expression, more recent functional and mechanistic studies have begun to establish direct connections between specific miRNAs and alterations in cell wall biosynthesis, lignification, and biomass traits (Zheng et al., 2019; Sharma et al., 2016; Zhang et al., 2021). Nevertheless, direct causal evidence connecting specific miRNAs to defined alterations in cell wall architecture, composition, or biomechanical properties remains limited and represents a critical area for future investigation.

One of the most compelling emerging directions is the recognition of miRNAs as mobile signaling molecules operating beyond traditional symplastic routes. Increasing evidence supports the role of EVs in mediating the transport of small RNAs through the apoplast, protecting them from degradation and enabling their delivery to distant cells or even across species boundaries (Baldrich and San Segundo, 2016; Cai et al., 2019). In this context, trans-kingdom RNA communication has gained considerable attention, as plant-derived miRNAs can be transferred to pathogens to regulate virulence-associated genes, while pathogen-derived small RNAs reciprocally modulate host pathways, including those involved in cell wall remodeling and defense, such as callose deposition (Cai et al., 2019; Huang et al., 2019). Fungal-derived siRNAs act as trans-kingdom effectors capable of crossing the plant cell wall and silencing host genes, including those associated with cell wall integrity and defense signaling, thereby modulating plasmodesmata dynamics and weakening structural barriers to facilitate infection. These findings redefine the plant cell wall not as a passive structural barrier but as a dynamic interface for RNA-mediated communication and signaling.

Beyond canonical miRNA-mediated pathways, emerging evidence suggests that endogenous small RNAs derived from cellulose synthase (*CESA*) antisense transcripts may constitute an additional regulatory layer controlling cell wall biosynthesis. These *CESA*-derived small RNAs appear to modulate cellulose synthase transcript abundance and broader wall-associated gene networks, potentially contributing to the transition between primary and secondary cell wall programs (Held et al., 2008; Nething et al., 2021). Future studies addressing the mobility, specificity, and integration of these regulatory RNAs with plasmodesmata-associated signaling pathways could provide new insights into RNA-mediated coordination of cell wall remodeling during development and stress responses.

Concurrently, high-throughput sequencing technologies continue to expand the landscape of miRNA biology, revealing numerous novel and stress-responsive miRNAs that remain functionally uncharacterized (Ryu et al., 2019; Rich-Griffin et al., 2020). This growing repertoire suggests that current models capture only a fraction of the regulatory complexity governing cell wall dynamics. Importantly, emerging functional evidence indicates that miRNAs influence not only gene expression but also the biomechanical properties of the cell wall. Regulatory modules such as miR397–laccase directly impact lignin biosynthesis, thereby modulating wall stiffness, integrity, and mechanical performance (Wang et al., 2014; Ali et al., 2023; Yang et al., 2024). These findings support a paradigm in which miRNAs act as integrators of molecular regulation and biomechanical outcomes.

From a technological perspective, the convergence of multi-omics, spatial transcriptomics, and single-cell approaches will be essential to resolve the cellular and tissue-specific dynamics of miRNA activity (Ryu et al., 2019; Rich-Griffin et al., 2020). Coupled with advanced imaging techniques, including super-resolution microscopy and live-cell tracking of fluorescently labeled miRNAs, these approaches will enable direct visualization of RNA mobility, elucidation of transport routes, and quantification of intercellular signaling dynamics (Skopelitis et al., 2018). In parallel, investigating EV-mediated transport mechanisms will provide new insights into how extracellular environments, including the cell wall matrix, influence RNA stability, mobility, and function (Baldrich and San Segundo, 2016; Cai et al., 2019).

From an applied perspective, manipulation of miRNA pathways represents a promising strategy for crop improvement. Advances in synthetic biology—including artificial miRNAs (amiRNAs), target mimics, and CRISPR-based genome editing—enable precise modulation of regulatory networks controlling cell wall composition (Tang and Chu, 2017). These approaches offer opportunities to optimize traits such as biomass production, stress tolerance, and postharvest quality. However, given the pleiotropic nature of miRNA regulation, such strategies must be carefully designed. Future efforts should prioritize tissue-specific and inducible expression systems, supported by comprehensive phenotypic, biomechanical, and multi-omics analyses to ensure that engineered modifications do not compromise plant fitness or developmental stability.

In summary, advancing this field will require a shift toward integrative, systems-level approaches capable of capturing the dynamic interplay between regulatory RNAs, cell wall architecture, and environmental responses. As summarized in **Figure 3**, miRNA biogenesis and intracellular regulation (**Figure 3A**), extracellular vesicle-mediated transport (**Figure 3B**), and their impact on cell wall remodeling and biomechanical properties (**Figure 3C**) collectively define a multiscale regulatory framework. In addition, emerging trans-kingdom RNA communication pathways (**Figure 3D**) further expand this regulatory landscape by linking plant and pathogen interactions. Bridging these dimensions will not only deepen our understanding of plant adaptive responses but also enable the rational design of crops with enhanced structural resilience and environmental fitness. Ultimately, emerging evidence supports a paradigm shift in which the plant cell wall is not merely a target of miRNA regulation, but an active participant in RNA-mediated signaling networks.Overall, understanding how miRNA-mediated regulatory networks coordinate cell wall dynamics, intercellular communication, and stress adaptation will be critical for advancing both fundamental plant biology and applied strategies for resilient crop systems.
